# The impact of health information technologies on quality improvement methodologies’ efficiency, throughput and financial outcomes: a retrospective observational study

**DOI:** 10.1186/s12911-016-0395-z

**Published:** 2016-12-05

**Authors:** Raed H. AlHazme, Syed S. Haque, Hal Wiggin, Arif M. Rana

**Affiliations:** 1 Department of Health Informatics, Rutgers University, School of Health Related Professions, Newark, NJ 07107 USA; 2 College of Osteopathic Medicine, Biomedical Informatics Program, Nova Southeastern University, Ft Lauderdale, FL 33314 USA; 3 College of Osteopathic Medicine, Public Health Program, Nova Southeastern University, Ft Lauderdale, FL 33314 USA

**Keywords:** Clinical and business intelligence, Data analytics, Healthcare challenges, Quality control, Quality improvement

## Abstract

**Background:**

To evaluate whether or not the utilization of Health Information Technologies (HITs) in Quality Improvement Methodologies (QIMs) has impacts on QIMs’ efficiency, throughput and financial outcomes at healthcare organizations and physician practices in the United States.

**Methods:**

This is a retrospective observational study that was conducted between the years of 2014 and 2015 and relied on two data sources: the Dorenfest Institute dataset and the Healthcare Information and Management Systems Society (HIMSS) Analytics data source. In addition, questionnaires were submitted to collect data about how healthcare settings in the United States had been utilizing QIMs in the last 10 years. The submitted questionnaire invitations yielded 144 responses from 134 hospitals and 10 physician practices. Descriptive statistics were used to assess the condition of the data. This involved the utilization of Box-Whisker plots to visualize the data shape, outliers and variation. The Gamma correlation analysis method was used to evaluate the statistical relationships between the QIM outcomes, efficiency, throughput and financial outcomes, and the employment of HIT systems in QIMs.

**Results:**

The study found that 99.3% of the healthcare organizations and physician practices had implemented at least one QIM over the last 10 years. In the QIM implementations, the total numbers of reported utilization instances of manual data collection, electronic health records, lab information systems, pharmacy information systems, computerized provider order entry and radiology information systems were 387, 352, 205, 185, 180 and 158, respectively. Based on a 95% confidence limit, the Gamma statistical test has shown an inverse correlation between the exclusive utilization of manual data collection and the overall QIM efficiency (*p* = 0.047, Gamma = −0.388) and throughput (*p* = 0.012, Gamma = −0.593) outcomes. However, the overall QIM financial outcomes were found to have a statistically insignificant correlation (*p* = 0.159).

**Conclusions:**

The study has revealed statistically significant negative impacts on QIMs’ efficiency and throughput outcomes when the manual data collection is the sole method used in QIM implementations. This also indicates a positive correlation between the QIMs’ efficiency and throughput outcomes and the HIT utilization in QIMs.

**Electronic supplementary material:**

The online version of this article (doi:10.1186/s12911-016-0395-z) contains supplementary material, which is available to authorized users.

## Background

Quality improvement is one of the key challenges facing healthcare organizations, health government agencies, and patient safety advocates [1, 2]. This issue has been highlighted in scientific and professional literatures. For instance, the Institute of Medicine (IOM) has reported that, in the United States, between 44,000 and 98,000 patients die due to preventable mistakes [3]. The IOM also estimated that around 1.5 million preventable medication errors occur each year in the United States [4]. From a cost perspective, it has been reported that the healthcare waste value in the United States has reached $750 billion in 2009 [5]. In Europe, the World Health Organization (WHO) reported that one of the healthcare quality issues, Health Care-Associated Infections (HCAIs), causes 16 million extra-days of hospital stay annually, resulting in financial losses of approximately € 7 billion every year (considering direct costs only [6]). Such serious alarms and others highlight the importance of implementing solutions that assist in overcoming quality of service challenges.

Over the years, multiple Quality Improvement Methodologies (QIMs) have been developed and employed in many industries. In fact, many healthcare organizations have already been utilizing a variety of those methodologies. A study that was conducted in the Netherlands has shown that 91% of responding hospitals have implemented at least one of the common methodologies [7]. The study also pointed that 39% of the hospitals have used five or more QIMs.

Despite their proven effectiveness [8–11], most QIMs were developed originally for work environments that were vastly different from healthcare. For example, the Lean methodology was developed by Toyota [12] and the Six Sigma methodology was mostly created by Motorola [13]. Both of these work environments are typically manufacturing-based. Even after a traditional QIM is extended to other industries, there are significant challenges in meeting the environment and other requirements of healthcare settings. Limitations [14, 15], resource demands [14, 16–18], inaccuracy [19] and impracticality [20] are some of the issues that have been highlighted in the literature. The significance of such issues is very high, and in fact, the reported high implementation failure rates of some of the traditional QIMs in healthcare [21] mostly results from these issues.

The previously mentioned QIM challenges are mainly related to the traditional data collection process, as data represent a key element in quality improvement [22]. The issues of the traditional data collection can be mitigated using the available information technology tools [23]; mainly Health Information Technology (HIT) systems. As of 2015, 56.7% of the surveyed 5462 hospitals in the United States are now using HIT systems to document patients’ workflows electronically from admission to discharge. This includes computerized provider orders entry and results [24]. In fact, the adoption of comprehensive HIT systems at United States non-federal hospitals has increased 11-fold since 2009 [25]. Considering this wide adoption, HIT systems have the potential to automate the data collection process in QIMs. However, it is uncertain whether or not the utilization of HIT systems can improve QIM efficiency, throughput and financial outcomes at healthcare organizations in the United States.

## Methods

This retrospective observational study started in January of 2014 and ended in April of 2015. The study relied on three data sources to evaluate healthcare organizations’ experiences of HIT utilization in QIMs over the last 10 years. The database provided by the Dorenfest Institute for Health Information was used to obtain demographical information about healthcare organizations. This included the organizations’ size, type, location, HIT status, and the contact information of the organization’s representatives. The Electronic Medical Record Adoption Model (EMRAM) of HIMSS was used as one of the data sources to attain information about whether or not the healthcare organization has reached a closed-loop HIT implementation, which involves capturing data from admission to discharge, including orders, results and medication administration. The “basic HIT installed” measure was obtained from the Dorenfest database, by identifying the three basic HIT applications for hospitals. The three - application benchmark has been set based on HIMSS EMRAM, which specifies that the basic installation of HIT consists of those three applications, as mentioned in stage 1 of the model [26]. However, in the case of physician practices, the only application that was checked is the “Ambulatory EMR” and the “Practice Management” systems in the Dorenfest database, which both represent Stage 1 of the HIMSS Ambulatory Electronic Medical Record Adoption Model [27]. It is important to highlight that “Practice Management” is a system that provides schedule management, patient demographics, medical billing management, claims scrubbing, and reporting capabilities [28].

The third data source was a questionnaire that evaluated the experiences of hospitals and practices in relation to HIT utilization in QIM. The questionnaire addressed the missing variables that were not provided by the Dorenfest Health Information database or HIMSS EMRAM, mainly in relation to the healthcare organizations’ experiences during QIM implementations. To obtain this information, an online questionnaire service was used to send survey invitations and collect the questionnaire responses. The instruments of the questionnaire can be referred to in the Additional file 1. The questionnaire included three key measures: efficiency, throughput and financial. The efficiency measure is defined as the performance of processes with the minimum resources required. The throughput measure is the number of processes that can be completed. The financial measure indicates the monetary cost needed to conduct a process.

The contact information of the surveyees were obtained from the Dorenfest Health Information database. The questionnaire invitations had only been submitted to specific job roles that are related to the study scope. The targeted job roles are C-suite, executives, clinical department heads, quality improvement specialists and management and medical informatics personnel.

The study focused on eight QIMs that have been proven effective [8–10]: Lean Six Sigma (LSS), Lean Management (LM), Six Sigma (SS), Clinical Pathways (CP), Business Process Reengineering (BPR), Continuous Improvement (CI), Total Quality Management (TQM) and Benchmarking.

The “hospital bed size” measure was derived from the hospital bed number. The values were categorized based on the Healthcare Cost and Utilization Project (HCUP) bed size categorization method [29]. The method takes into consideration the hospital’s teaching status as well as metropolitan and geographical location aspects.

To determine the metropolitan status of hospitals, the 2010 United States Census Bureau classification for urban and rural areas was used [30]. The United States Census Bureau’s urban-rural classification defines geographical areas, identifying both the individual urban areas and the rural areas of the United States. The Bureau’s urban areas represent densely developed territory, and encompass residential, commercial, and other non-residential urban land uses. Rural areas encompass all population, housing, and territory not included within an urban area.

The United States region measure was derived from the United States Census Bureau regional divisions [31], which divide the country into four regions: Northeast, Midwest, South and West.

### Statistical methods

SPSS version 22.0.0.0 software was used to conduct the statistical analyses. Descriptive statistics was used to assess the condition of the data. This involved the utilization of Box-Whisker plots to visualize the data shape, outliers and variations. The Gamma correlation analysis method was used to evaluate the statistical relationship between the QIM outcomes, efficiency, throughput and financial outcomes, and the employment of HIT systems in QIMs.

## Results

The submitted questionnaire invitations yielded 144 responses from 134 hospitals and 10 physician practices, representing 2.3% of the 5723 hospitals that exist in the United States [32]. The locations and teaching statuses of the participating hospitals are classified as 50% urban non-teaching, 38.1% rural and 11.9% urban teaching (Table 1).

**Table 1:** Descriptive Summary of the Participating Healthcare Organizations (*n* = 144)

Organization Type	*n* (%)
Hospital	134 (93.1)
Ambulatory	10 (6.9)
Location and Teaching Status	
Rural	51 (38.1)
Urban, nonteaching	67 (50.0)
Urban, teaching	16 (11.9)
Region	
Northeast	35 (24.3)
South	48 (33.3)
Midwest	40 (27.8)
West	21 (14.6)
Organization’s Size	
Small Hospital	45 (33.6)
Medium Hospital	20 (14.9)
Large Hospital	69 (51.5)
Basic HIT Implemented	
Yes	141 (97.9)
No	3 (2.1)
Comprehensive HIT Implemented (Hospitals)	
Yes	37 (27.6)
No	97 (72.4)

**Fig. 1:**
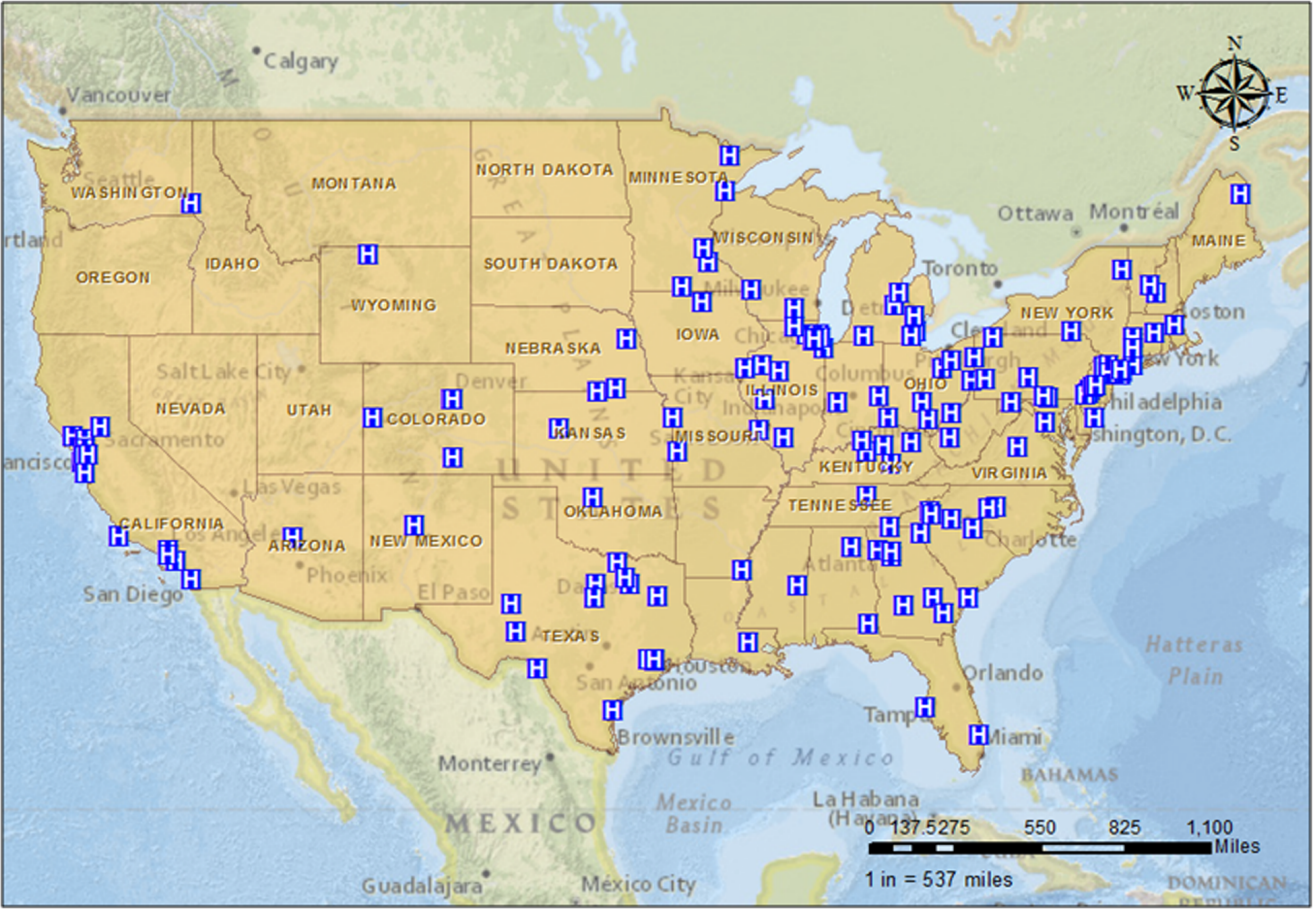
The responses from hospitals and physician practices have covered the four main regions of the United States

The geographical locations of the responding healthcare organizations included the four main regions of the United States, as defined by the United States Census Bureau. The participation shares of the Northeast, Midwest, South, and West regions were 24.3, 27.8, 33.3 and 14.6%, respectively. Figure 1 illustrates the geographical locations of the healthcare organizations that responded to the questionnaire.

**Fig. 2:**
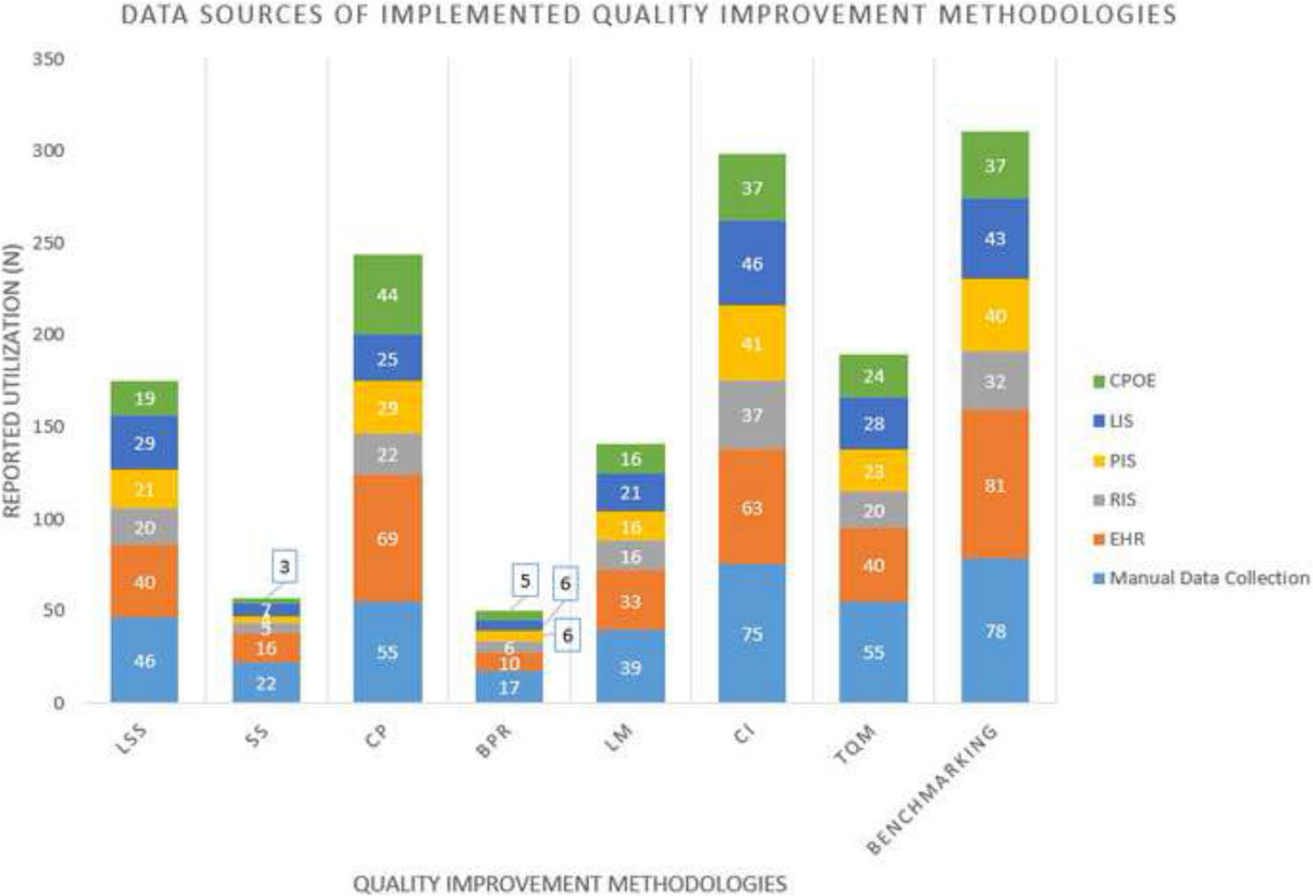
Data sources of the implemented QIMs

The QIM data sources used during the implementations in the participating healthcare organizations are presented in Fig. 2. It is important to highlight that in many cases, multiple data sources had been used by the healthcare organization as part of a QIM implementation. It is also imperative to mention that ToC had a very low number of reported utilization of data sources; one manual data collectin and one CPOE. Therefore, this QIM was removed from Fig. 2 in order to improve the appearance of the other significant categories in the graph.

Although several types of HIT systems had been used as data sources in many of the QIM implementations, manual data collection was the most common method, with the highest utilization score in six out of the eight QIMs and a total number of 387 reported utilization instances. It was also observed that the Electronic Health Record (EHR) system was the second most common data collection method in QIM implementations. The EHR scored the second highest utilized data source in six out of the eight QIMs, while it ranked the highest utilized in the remaining two. The total number of EHR utilization cases was found to be 352. This is higher than the utilization instances total number of Lab Information Systems (LIS’s), Computerized Provider Order Entry (CPOE), Pharmacy Information Systems (PIS’s) and Radiology Information Systems (RIS’s), in which they had total utilization numbers of 205, 185, 180 and 158, respectively.

The reported overall outcomes of all main QIMs were found to be at around the middle level in all three outcome areas: efficiency, throughput, and financial improvement. The majority of the Standard Deviation (STD) values were below one, which indicated that the variations in the outcome data were low, and that most of the reported outcome values were generally at the moderate level. Table 2 displays the overall outcomes of each of the common QIMs that were included in the study.

**Table 2:** Overall outcomes of QIM implementations

	Efficiency of Workflow $\overline{x}$(STD)*	Throughput of Workflow $\overline{x}$(STD)*	Financial Improvement $\overline{x}$(STD)*
Lean Six Sigma (LSS)	3.72 (0.86)	3.7 (0.86)	3.45 (1.1)
Six Sigma (SS)	3.88 (0.83)	3.68 (0.98)	3.62 (1.05)
Clinical Pathways (CP)	3.8 (0.82)	3.74 (0.84)	3.25 (1.06)
Business Process Reengineering (BPR)	3.45 (0.82)	3.42 (0.9)	3.17 (0.94)
Lean Management (LM)	3.3 (0.96)	3.25 (0.96)	3.36 (1.0)
Continuous Improvement (CI)	3.54 (0.93)	3.51 (0.84)	3.23 (1.02)
Total Quality Management (TQM)	3.33 (1.03)	3.28 (0.97)	3.0 (1.02)
Benchmarking	3.54 (0.9)	3.57 (0.91)	3.28 (1.0)
Average	3.57 (0.89)	3.52 (0.91)	3.3 (1.02)

*The used measurement scale is from 0 to 6, which was averaged in the table

In order to explore the influence of HIT utilization on QIMs, the dataset was filtered to eliminate the observations that did not have a complete reliance on the HIT data sources. The results have shown noticeable differences in the outcomes between the two groups; the QIM implementations that relied on all data sources, Table 2, and the HIT-based QIM implementations, Table 3.

**Table 3:** Outcomes of HIT-based QIM implementations

	Efficiency of Workflow $\overline{x}$(STD)*	Throughput of Workflow $\overline{x}$(STD)*	Financial Improvement $\overline{x}$(STD)*
Lean Six Sigma (LSS)	4.2 (0.84)	4.2 (0.84)	3.6 (1.14)
Six Sigma (SS)	4.0 (0.00)	3.33 (0.58)	3.67 (0.58)
Clinical Pathways (CP)	4.13 (0.64)	4.0 (0.54)	3.63 (1.19)
Business Process Reengineering (BPR)	N/A	N/A	N/A
Lean Management (LM)	3.86 (1.07)	3.71 (0.95)	2.86 (1.22)
Continuous Improvement (CI)	4.0 (0.63)	4.33 (0.54)	3.33 (1.21)
Total Quality Management (TQM)	3.83 (0.75)	3.67 (0.52)	3.17 (0.98)
Benchmarking	3.67 (0.5)	3.6 (0.53)	3.89 (0.6)
Average	3.96 (0.74)	3.83 (0.64)	3.45 (0.99)

*The measurement scale is from 0 to 6, and the averages are in the table

The results of the descriptive statistics, revealed in Tables 2 and 3, have shown that the mean values for efficiency, throughput and financial outcomes were 3.57, 3.52 and 3.3. Correspondingly, they were lower compared to QIM implementations that involved the exclusive use of HIT systems, 3.96, 3.83 and 3.45. It was also important to highlight that there was a very high consistency in the reported outcomes, as the STDs were lower than one on all outcomes.

### Throughput outcomes

To further evaluate the impact of the manual data collection practice on throughput outcomes, correlation analysis was performed on the variable “Only Manual Data Collection Was Used”. This flagged the observation when QIM was implemented without relying on HIT data sources, and the variable that showed the QIM implementation throughput outcomes, “Average Throughput Outcome”.

**Fig. 3:**
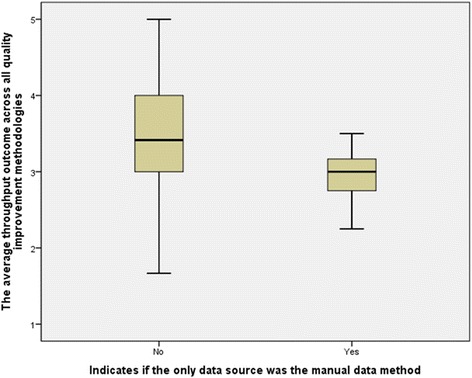
Box-Whisker plot for the impact of manual data collection on throughput outcomes of QIMs

The results of the basic statistics for the two groups; the group that relied completely on manual data collection in QIM implementations and the group that did not, have revealed a number of findings. Both, the throughput outcome mean and median were lower for the group that exclusively utilized the manual data collection method in QIM, 2.95 and 3.00, compared to 3.48 and 3.41 for the group that used HIT data sources. The Box-Whisker plot, presented in Fig. 3, also confirms the findings that were revealed through the descriptive statistics. Although the group that did not rely exclusively on the manual data collection method had a wider value range than the group that did, its median, 1st quantile and 3rd quantile were higher.

The correlation result of the Gamma test shows a statistically significant association between the two variables, *p*-values = 0.012 (95% Confidence Limit). The direction of the correlation analysis demonstrated an inverse, or negative, correlation between the manual data collection and throughput outcomes, Gamma = −0.593, which also indicated a moderate degree of inverse correlation. This reveals that when manual data collection is used, throughput outcomes of QIMs decrease and vice versa. Additionally, it implies that the utilization of HIT systems in QIMs improve throughput outcomes, because the absence of the manual data collection method entails the utilization of HIT systems as data sources.

To identify the QIMs that yielded throughput improvements when HIT systems are used, correlation tests have been performed on all of the selected common QIMs. However, the only QIMs that had a statistically significant correlation with the utilization of HIT systems were LSS and CP.

The Gamma correlation test between the manual data collection and LSS throughput outcomes yielded a *p*-value of 0.032, which suggests rejecting the null hypothesis *(H*_*0*_ *)* and accepting the alternative hypothesis *(H*_*A*_ *)*, based on 95% confidence limit. This points to the statistically significant correlation between the manual data collection and throughput outcomes of LSS implementations. The direction and strength of the correlation is indicated by the Gamma value, −0.505, which points to a moderate negative correlation. This suggests that there is statistically significant evidence that throughput outcomes go down when the manual data collection method is used solely in the implementation of LSS and vice versa.

The correlation test of CP throughput outcomes and the utilization of HIT systems resulted in a significance value of 0.01, suggesting the rejection of the null hypothesis *(H*_*0*_ *)* and the acceptance of the alternative hypothesis *(H*_*A*_ *)*, based on a 95% confidence limit. This infers to the statistically significant correlation between the manual data collection and throughput outcomes of CP implementations. The direction and strength of the correlation is specified by the Gamma value, −0.45, which points again to a moderate negative correlation. This proposes that there is a statistically significant evidence that throughput outcomes decrease when the manual data collection method is used solely in the implementation of CP and vice versa.

### Efficiency outcomes

In relation to efficiency outcomes, descriptive statistics revealed that the efficiency outcome mean and median were lower for the group that exclusively utilized the manual data collection method in QIM, 3.10 and 3.17, compared to 3.48 and 3.50 for the group that utilized HIT data sources. The data variation in both groups was very minimal, as the STD values were found to be below 1 in both groups.

**Fig. 4:**
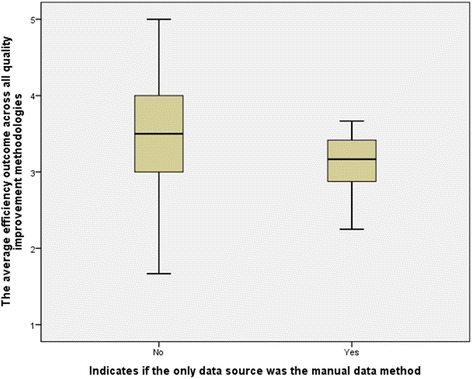
Box-Whisker plot for the impact of manual data collection on efficiency outcomes of QIMs

Figure 4 is a Box-Whisker plot that shows the impact of manual data collection on efficiency outcomes of QIMs. The plot also confirms the findings that were revealed through the descriptive statistics. Although the group that did not rely exclusively on the manual data collection method had a wider value range than the group that did, its median, 1st quantile and 3rd quantile were higher.

The efficiency outcomes variable also has shown a statistically significant association with the manual data collection method. The *p*-value was 0.047, lower than 0.05 based on 95% confidence limit. Therefore, the null hypothesis (*H*_*0*_ *)* was rejected and the alternative hypothesis (*H*_*A*_) was accepted. The correlation was found to be inverse with moderate strength, Gamma = −0.388, suggesting that the overall efficiency outcomes decrease when the manual data collection method is utilized in QIMs.

However, when each QIM efficiency outcome variable was tested individually against the variable of the manual data collection method, the only QIMs that showed statistically significant correlations were LSS and CP.

The correlation between the manual data collection method and LSS efficiency outcomes has been found to be statistically significant. With the *p*-value = 0.035, lower than 0.05 based on 95% confidence limit, the null hypothesis (*H*_*0*_ *)* was rejected and the alternative hypothesis (*H*_*A*_) was accepted. The correlation was found to be moderate with an inverse direction, Gamma = −0.494, suggesting that the LSS efficiency outcomes decrease when the manual data collection method is utilized. This assumption also inversely applicable on the utilization of HIT systems in LSS implementations, as not using the manual data collection exclusively would imply the utilization of one or more HIT systems as data sources in the LSS implementation. Consequently, the finding here suggests that the utilization of HIT systems would have a positive impact on LSS efficiency outcomes.

For CP efficiency outcomes and the utilization of the manual data collection method, the significance test of the CP efficiency outcomes and manual data collection suggested that there is a correlation between the two variables. The *p*-value was found to be 0.007, lower than 0.05 based on 95% confidence limit. Therefore, the null hypothesis (*H*_*0*_ *)* was rejected and the alternative hypothesis (*H*_*A*_) was accepted. The Gamma value has shown a strong moderate negative correlation, Gamma = −0.456, suggesting that the CP efficiency outcomes decrease when the manual data collection method is utilized in QIMs.

### Financial outcomes

The association between the average financial outcomes and the manual data collection method was also tested with correlation analysis. Descriptive statistics revealed that both, the financial outcome mean and median were lower for the group that exclusively utilized the manual data collection method in QIM, 3.03 and 3.00, compared to 3.25 and 3.25 for the group that utilized HIT data sources. Moreover, the data variation in both groups was very minimal, as the STD values were found to be below 1 in both groups. Besides the close mean and median values, the findings indicate that the data within the groups were relatively normally distributed.

**Fig. 5:**
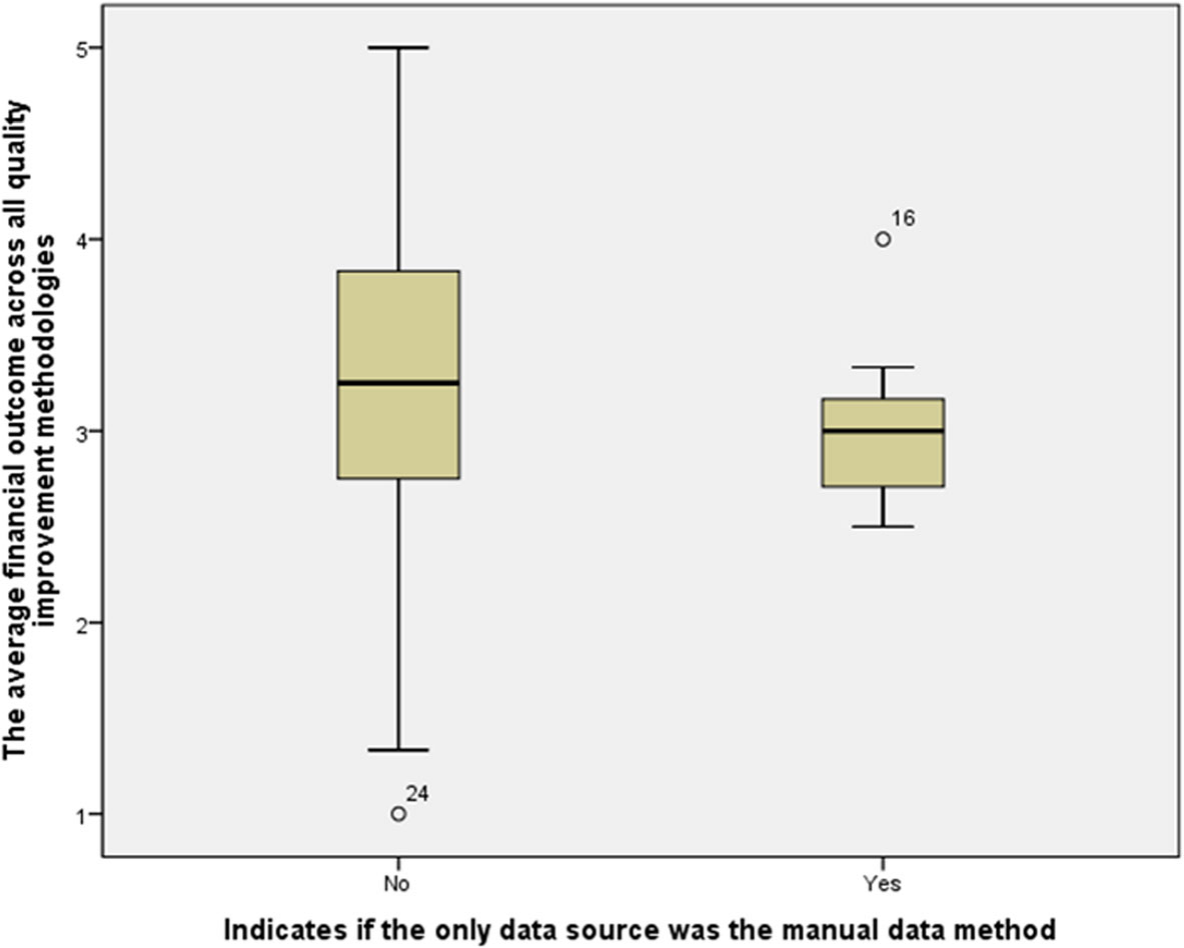
Box-Whisker plot for the impact of manual data collection on financial outcomes of QIMs

Box-Whisker plot, Fig. 5, shows the impact of manual data collection on financial outcomes of QIMs. The plot also confirms the findings that were revealed through the descriptive statistics. Although the group that did not rely exclusively on the manual data collection method had a wider value range than the group that did, its median, 1st quantile and 3rd quantile were higher.

However, the overall financial outcome variable did not show a statistically insignificant association with the manual data collection. The *p*-value was 0.159, which suggests accepting the *H*_*0*_, based on 95% confidence limit. Nonetheless, CP was the only QIM that was found to individually have a statistically significant correlation with the financial outcomes when HIT systems are used as data sources.

The Gamma correlation test showed that the financial outcome variable had a statistically significant association with the manual data collection method. The *p*-value was 0.015, lower than 0.05 based on 95% confidence limit. Therefore, the null hypothesis (*H*_*0*_ *)* was rejected and the alternative hypothesis (*H*_*A*_) was accepted. The correlation was also inverse at a moderate level, Gamma = −0.4, suggesting that the overall efficiency outcomes decrease when the manual data collection method is utilized in QIMs.

## Discussion

Although the study has found that the manual data collection method negatively affects QIM efficiency outcomes, it is unclear whether or not an automatic data collection method, based on HIT, can show a positive correction. A further study should evaluate this hypothesis by developing a HIT-integrated QIM module that collects the data directly from the HIT systems. The module should then be implemented and studied to evaluate the hypothesis.

LSS, one of the key QIMs [8, 9] that has been proven to be effective [33, 34], can be used as the QIM that will be implemented in the prototype. LSS consists of two main parts: Lean and Six Sigma. Lean, which was largely developed by Toyota Motor Corporation [12], is designed around the customer requirements to ensure the delivery of products or services in the most effective, timely and safe manner possible [35]. Six Sigma is mainly about reducing variations in processes. Both of these parts can benefit from the implemented HIT systems in healthcare organizations. Nowadays, many healthcare organizations in the United States are capable of capturing the necessary LSS data through HIT systems. According to HIMSS, 52.9% of the surveyed 5458 hospitals in the United States are now using HIT to electronically document patients’ workflows from admission to discharge, including the different orders and results that get placed and documented during the encounter [36]. This current high level of HIT adoption makes the HIT-integrated LSS module relevant to a majority of the healthcare organizations. In fact, the average adoption rates of basic EHRs in the last 4 years [37] indicates that the adoption percentage will reach 90% in the next few years.

**Fig. 6:**
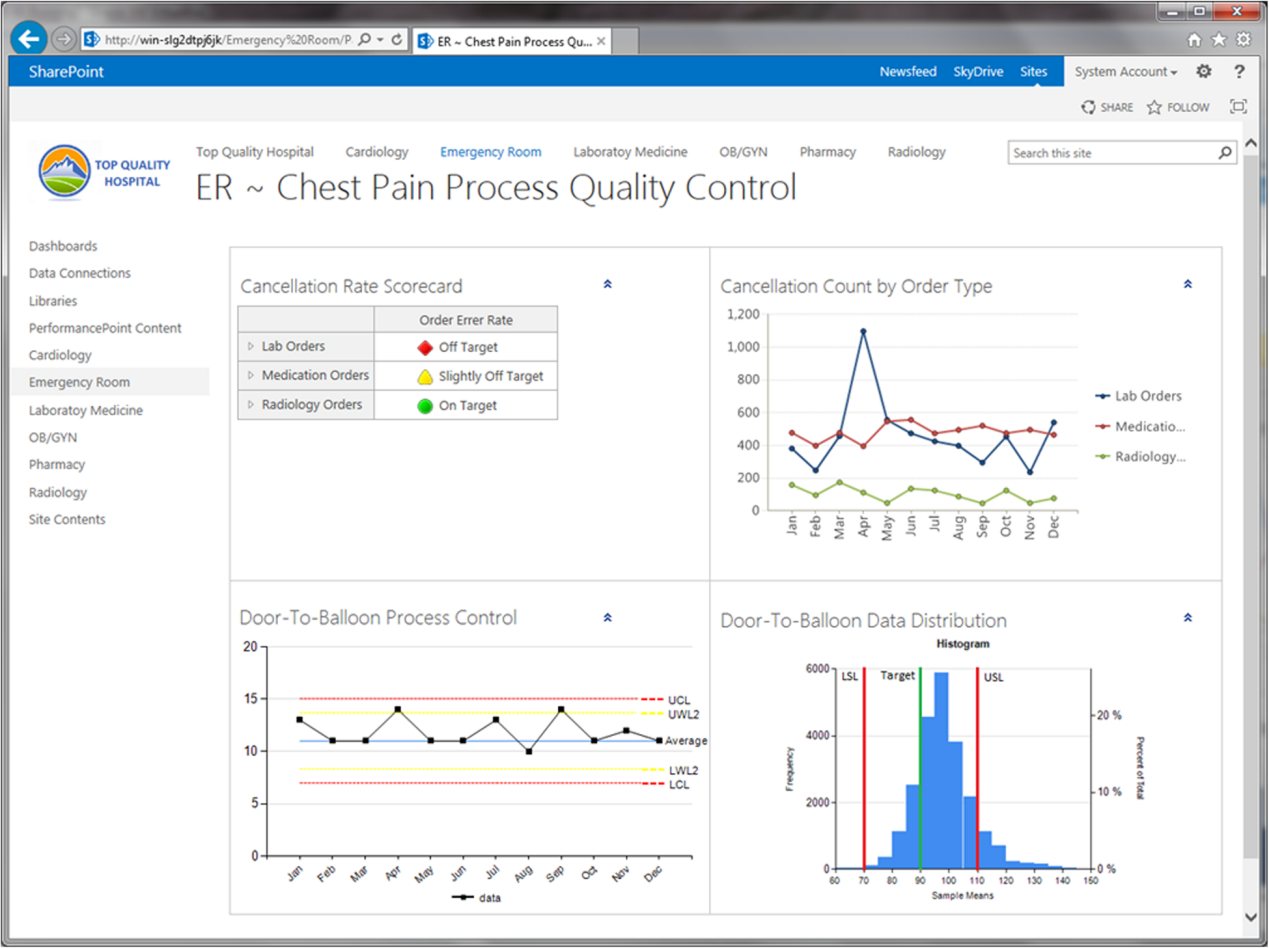
Dashboards can help quality improvement teams maintain positive changes

An HIT-integrated LSS module can be developed based on a Clinical and Business Intelligence (CBI) framework. While HIT systems deliver the clinical data, CBI automates the implementation of the various LSS tools. This can be facilitated through the different CBI components, including the data transformation, analysis and presentation functionalities. After the data is formatted properly for analytics, CBI tools can be configured to perform LSS analyses for the Define, Measure, Analyze, Improve and Control (DMAIC) phases. For instance, the dashboard component of the module can facilitate the control phase and help the quality improvement team members maintain the positive changes over the long-term. Figure 6 shows an example of the module’s dashboard.

### Study limitations and future research

The study was limited to evaluating QIM experiences based on general understanding of the QIMs’ definitions, processes and implementation methods. However, these elements are not well established and standardized in the quality improvement field and this results in variations among QIM implementations. To avoid the implementation variation issue and to better control the constant variables, a cohort study is recommended to evaluate the outcomes of QIM with and without utilizing HITs as data sources.

## Conclusions

The healthcare system in the United States is faced with major quality challenges. QIMs have been proven to be effective in many industries. Nevertheless, it is important to implement QIMs in a manner that suites the healthcare environment and integrates with its processes. HIT, with its current wide deployment, can provide an ideal platform that integrates QIMs into healthcare workflows. However, it was unclear whether the reliance on HIT systems as data sources can improve QIM outcomes. This study has found that 99.3% of the healthcare organizations have implemented at least one of the common QIMs mentioned in the questionnaire. It was also revealed that collecting quality improvement data manually was the most common technique used in QIMs. The study has identified statistically significant negative impacts on QIMs’ efficiency and throughput outcomes when the manual data collection is the sole method used in QIM implementations. It also indicates a positive correlation between the QIMs’ efficiency and throughput outcomes and the utilization of HIT systems in QIM implementations.

## Additional file

Additional file 1: Survey instruments. (PDF 242 kb)

## Abbreviations

BPR: Business process reengineering
CBI: Clinical and business intelligence
CI: Continuous improvement
CP: Clinical pathways
DMAIC: Define measure, analyze, improve and control
EHR: Electronic health record
EMRAM: Electronic medical record adoption model
HCAI: Health care-associated infection
HCUP: Healthcare cost and utilization project
HIMSS: Healthcare information and management systems society
HIT: Health information technologies
IOM: Institute of medicine
LM: Lean management
LSS: Lean Six Sigma
QIM: Quality improvement methodologies
SS: Six Sigma
STD: Standard deviation
TQM: Total quality management
WHO: World health organization
