# Supplementary material for: The impact of health information technologies on quality improvement methodologies’ efficiency, throughput and financial outcomes: a retrospective observational study
Source: BMC Med Inform Decis Mak. 2016 Dec 5;16:154. doi: 10.1186/s12911-016-0395-z (PMC5139044; doi:10.1186/s12911-016-0395-z)
Supplement: Additional file 1: — Survey instruments. (PDF 242 kb) [file 12911_2016_395_MOESM1_ESM.pdf]

Additional File 1: Survey Instruments

Survey Title: *The Impact of Health Information Technologies on Quality Improvement Methodologies' Efficiency, Throughput and Financial Outcomes: A Retrospective Observational Study*

Question 1

Has the hospital/practice ever implemented any quality improvement methodology in the past?

Quality improvement methodologies include any technique that targets clinical or business processes for improvement. Examples: Clinical Pathways, Benchmarking, Lean Six Sigma, Total Quality Management, Continues Improvement, etc...

( ) Yes

( ) No

Question 2 of 2

**Logic: Hidden unless: Question "Has the hospital/practice ever implemented any quality improvement methodology in the past?" #1 is one of the following answers ("No")**
Variable name: Array

What are the reasons for not implementing quality improvement methodologies?

[ ] No quality improvement resources are available to conduct quality improvement

[ ] No financial demands exist to conduct quality improvement at this point

[ ] No regulatory demands require the hospital/practice to implement quality improvement at this point

[ ] There is no time available to allocate for quality improvement

[ ] Quality improvement is generally not needed to be implemented in the hospital/practice at this point

[ ] other: ____

Question 2 of 7

**Logic: Hidden unless: Question "Has the hospital/practice ever implemented any quality improvement methodology in the past?" #1 is one of the following answers ("Yes")**
Variable name: Array

What are the quality improvement methodologies that have been used at the hospital/practice over the last ten years?

[ ] Clinical Pathways (CP)

[ ] Benchmarking

[ ] Lean Six Sigma (LSS)

[ ] Six Sigma (SS)

[ ] Business Process Reengineering (BPR)

[ ] Lean Management (LM)

[ ] Theory of Constraints (ToC)

[ ] Continuous Improvement (CI)

[ ] Total Quality Management (TQM)

[ ] other: ____

Question 3 of 7

**Logic: Hidden unless: Question "Has the hospital/practice ever implemented any quality improvement methodology in the past?" #1 is one of the following answers ("Yes")**

In what period of time the quality improvement methodologies have/had been used?

	Last 12 Months	Between 1 and 5 Years Ago	Between 6 and 10 Years Ago

---

Question 4 of 7

Logic: Hidden unless: Question "Has the hospital/practice ever implemented any quality improvement methodology in the past?" #1 is one of the following answers ("Yes")

For each of the quality improvement methodologies selected previously, from where the data came?

	Manual Data Collection	Electronic Health Record	Radiology Information System	Pharmacy Information System	Lab Information System	CPOE

Question 5 of 7

Logic: Hidden unless: Question "Has the hospital/practice ever implemented any quality improvement methodology in the past?" #1 is one of the following answers ("Yes")

Have the goals of the implemented quality improvement methodology(s) been achieved? Based on the efficiency, throughput, and financial factors.

	Efficiency of Workflow	Throughput of Workflow	Financial Improvement

---

Question 6 of 7

Logic: Hidden unless: Question "Has the hospital/practice ever implemented any quality improvement methodology in the past?" #1 is one of the following answers ("Yes")

Variable name: Array

If you have previously answered that manual data collection has been used, what are the reasons for collecting the quality data manually instead of retrieving the data from clinical systems (EHR, CPOE, LIS, etc...)?

[ ] Clinical systems did not have the needed data elements

[ ] Did not have access to the databases of the clinical systems

[ ] It was easier to collect the data manually

[ ] There were technical challenges in retrieving the data from systems

[ ] Quality improvement methodologies require manual data collection

[ ] We do not have clinical systems implemented at the site

[ ] Others: ____

Question 7 of 7

Logic: Hidden unless: Question "Has the hospital/practice ever implemented any quality improvement methodology in the past?" #1 is one of the following answers ("Yes")

Variable name: Array

Separately or as part of quality improvement methodologies, what are the quality improvement tools that have been used by the hospital/practice?

[ ] Process Capability Analysis

[ ] Statistical Process Control

[ ] Failure Mode and Effects Analysis (FMEA)

[ ] Cause-and-Effect Diagram (Fishbone or Ishikawa)

[ ] Supplier, Input, Process, Output, and Customer (SIPOC or COPIS)

[ ] System of Work (SOW)

[ ] RUMBA

[ ] SMARTI

[ ] X - Y Matrix

[ ] Process Failpoint Analysis Matrix

[ ] Waste Analysis Matrix

[ ] Five Whys

[ ] Process Mapping

[ ] SWOT Analysis

[ ] SCAMPER Analysis

[ ] other: ____
